# An evaluation of registered nurses’ experiences of person-centered care and competence after participating in a course in digital competence in care

**DOI:** 10.1186/s12912-022-01151-2

**Published:** 2022-12-23

**Authors:** Malin Carlsson, Annika Kjällman Alm, Malin Holmström Rising

**Affiliations:** Department of Health Sciences, Mid Sweden University, Holmgatan 10, Sundsvall, 852 33 Sweden

**Keywords:** Competence, Digital care, Person-centred care, Qualitative content analysis, Registered nurses

## Abstract

**Background:**

Health care’s rapid transition from in-person visits to more digital care meetings has challenged nurses to find new, sustainable ways of using digital technology.

**Methods:**

The aim was to describe registered nurses’(RN) experiences with person-centred care (PCC) and competence after participating in a course in Digital Competence in Care (DCC). In this study, a qualitative descriptive design was used, and 16 individual interviews were carried out with RNs. Data were analysed using qualitative content analysis. The COREQ checklist was used in this study.

**Results:**

The results were presented in four categories: being open to change and new ways of working with patients; struggling to handle requirements; developing new ways of working and focusing on patients despite the distance.

**Conclusions:**

The DCC course helped develop RNs' skills and practice of PCC in digital care meetings. Training in digital care theory increased RNs' competence and facilitated the creation of new knowledge. The RNs' professional role was strengthened by participating in the changing of work routines. Digital care meetings were shown to be distance bridging and complementary to physical care meetings contributing to PCC. The increased availability of health care via digital means has affected the consumption of care and tailored education needs for RNs must be met by nursing education programs. Digital care is accessible, efficient and enables care regardless of geographical conditions, its innovative development needs to be based on science and experience and RNs are key personnel in this process.

**Trial registration:**

Not applicable.

**Supplementary Information:**

The online version contains supplementary material available at 10.1186/s12912-022-01151-2.

## Background

Health care is a knowledge-intensive sector currently experiencing a paradigm shift with a change from physical, in-person care to more long-distance care via digital technology. The country’s growing and ageing population and its diminishing financial and human resources have created gaps between what is available and what is needed [1]. In response, groups in Sweden have called for health care reform, with the inclusion of PCC as one of the core competences in health care [2]. The reform also includes more digital services in health care [3, 4]. Likewise, the Swedish government’s vision of the digitalisation of health care [5] expects that by 2025, Sweden will offer high-quality, equitable health care and welfare services and be established as a world leader in eHealth—that is, a modernised form of health care delivered digitally [4, 6]. According to the World Health Organization [7], the importance of eHealth will only increase with time, especially as digital technology is increasingly used to treat patients, conduct research and education, track diseases, and monitor public health. One component of eHealth known as digital care meetings involves digital contact between patients and health care professionals via video meetings [8]. With the same goals as physical visits, digital care meetings facilitate patient–professional communication by using imaging technology that allows the participants to see and talk to each other in real time. In that process, digital competence is needed and will strengthens a person’s ability to interact with others digitally in consideration of everyone’s opportunities, rights, and obligations [9].

RNs should not only be digital competent themselves, but they should also contribute to developing the digital management of information while providing secure, high-quality digital care but also work in person-centred ways and strengthen patients’ digital competence [10–12]. This means that RNs' need to adapt their work processes in digital care meetings to establish relationships with patients and promote patients’ participation in the care in order to work in person-centred ways [13, 14]. To be sure, as digital technology has continued to alter approaches to working, the need to acquire and assimilate new skills has arisen [15]. To keep up with the tide of eHealth, RNs need to be flexible, patient, and capable of handling technical changes and challenges [16]. They also need to be able to combine traditional clinical approaches with new digital approaches [15].

Health care’s rapid transition from in-person visits to more digital care meetings has challenged RNs to find new, sustainable ways of using digital technology. To date, studies have overwhelmingly focused on the technical aspects of eHealth, meaning that more information from RNs' perspectives is needed for a deeper understanding of their competence needs.

### Aim

Therefore, the aim of this study was to describe RNs experiences with PCC and competence after participating in a course in DCC.

## Methods

### Design

A qualitative descriptive design with an inductive approach was chosen to gain a deeper understanding of RNs' experiences with PCC and competence in digital care meetings after participating in a DCC course [17]. The COnsolidated criteria for REporting Qualitative research (COREQ) checklist (Appendix S1) were followed in the study [18].

### Context

The study was performed in primary healthcare in a region in central Sweden. This region has a population of 245,000 inhabitants [19] and contains both rural and urban areas.

### Procedure

An online course, DCC was developed in collaboration with RNs from various health care settings in the region. The DCC course consisted of five units with content relevant to RNs' digital competence, including PCC, Gamification through an application with a digital game (The Person-Centred Game) focusing on PCC was also used in the DCC course [20]. To enable participation despite RNs' irregular working hours it was emphasised that the course must be. Flexible and online with no physical meetings, recorded lectures (films) and a study guide with reading instructions for each section. All course material were always available for the participants. The starting point was from the participant’s own prior experiences and issues related to their work and the course was designed and conducted with a curriculum of seminars for each section. The participants prepared themselves prior to the seminars with the course literature, research articles and their own experiences. At the online seminars all participants met to share and exchange experiences, learn from each other, and to start networking. The DCC course was designed from the concepts Bring-Your-Own-Data (BYOD1)—work with real and authentic problems or issues that the participants identify themselves through their work or activity and Bring-Your-Own-Device (BYOD2)—participants use equipment (computer etc.) from work (organization owned) [21]. The DCC course was also inspired by Learning by doing [22] and participants worked together and shared experiences from authentic problems in seminars. The DCC course was given as five four-hour seminars (a total of 20 h) across ten weeks. An overview of the content in the course is shown in Table 1.

**Table 1:** An overview of the content of the DCC course

Week	Seminars in zoom (4 h)	Content	The person-centred- game
1	1	eHealth and PCC	Level 1–3
3	2	Digital documentation in medical records	Level 4
6	3	Digitized care meetings with patients	Level 5–6
8	4	Pedagogy, ethics, legislation and law in digitalized care	Level 7–8
10	5	Remote digital care, and organizational development	Level 9

### Participants

A purposive sample was used in this study, ten RNs participated in the DCC course in March 2020, eight RNs agreed to participate in individual interviews after completing the course. Two RNs did not participate in the interviews due to COVID-19 pandemic [17]. The participants were provided with written information regarding the study by one of the authors. Once the consent form was returned to the authors, interviews were arranged and undertaken at a time convenient for the participants. The participants were all women who were 38–62 years old (median = 49 years) and had worked as RNs for 16–40 years (median = 25,5 years). All participants (n = 8) were specialist RNs with a master’s degree in nursing. The study took place in primary healthcare. The sample’s characteristics and the data collection process are shown in Table 2.

**Table 2:** Sample characteristics and data collection

Participants	Gender	Age	Education	Work experiences	Interview 1	Interview 2
R**N** 1	F	42 years	MSc in Nursing	16 years	56 min	72 min
RN 2	F	51 years	MSc in Nursing	26 years	61 min	59 min
RN 3	F	38 years	MSc in Nursing	18 years	55 min	39 min
RN 4	F	47 years	MSc in Nursing	25 years	63 min	47 min
RN 5	F	52 years	MSc in Nursing	28 years	67 min	56 min
RN 6	F	62 years	MSc in Nursing	40 years	79 min	60 min
RN 7	F	56 years	MSc in Nursing	30 years	50 min	55 min
Rn 8	F	45 years	MSc in Nursing	21 years	65 min	58 min
		Median = 49 years		Median = 25,5 years	Median = 62 min	Median = 57 min
Total time for Interviews I and II					496 min	438 min
Total time for all interviews					934 min or 15 h and 34 min	

### Interviews

Data were collected through interviews, which took place by telephone due to the pandemic, by the first and last authors at two occasions, two months (in June 2020) and ten months (in February 2021) after the DCC course. Each participant was interviewed twice and in total, 16 individual interviews were conducted. The first author conducted 10 interviews (both occasion 1 and 2) and the last author conducted 6 interviews (both occasion 1 and 2). The interviews were performed in Swedish with in-depth open-ended questions. The same interview guide was used in all interviews to maintain focus, and the main questions addressed were as follows: Can you tell me about your experiences regarding your own competence after participating in the DCC? Can you tell me about your experiences with PCC after participating in the DCC? Can you describe your daily work with digital care meetings and PCC? Follow-up questions (e.g., “Can you tell me more about that?”) were asked as necessary. The first interviews lasted for 50–79 min (median = 62 min), and the second interviews lasted for 39–72 min (median = 57 min). The total sample consisted of 934 min or 15 h and 34 min, and the audio recordings were transcribed verbatim by all authors [17] (Table 2).

### Data analysis

Following Graneheim and Lundman [23], inductive content analysis was used to discover patterns, subcategories, and categories in the text. The analysis involved several steps, beginning with reading the transcripts from both the first and second interviews in their entirety. The initial reading offered an overview of what the different interviews focused on and an overarching sense of the different components of the content [23]. After that, the transcripts from the first and second interviews were analysed separately. After the first reading of the transcripts, each transcript was read again in its entirety and divided into units of meaning according to the aim of the study identified from part to part. Next, the meaning units were condensed into a description close to the text, the manifest content, and labelled with a code relevant to the purpose of the study. The condensed meaning units and the related codes were abstracted and sorted into subcategories depending on their similarities and differences. The structures that emerged as a result enabled the identification and formation of categories from the subcategories. Last, all interviews were woven together to reach a comprehensive understanding of RNs experiences with PCC and competence. To ensure that no meanings were overlooked, the findings were compared and validated with the original material [23]. All authors participated in the analysis. An example of the analysis is shown in Table 3.

**Table 3:** Example of the analysis process

Meaning units	Condensed statements	Codes	Subcategories	Categories
Digital competence involves daring to deal with new digital systems and to challenge myself and [my] coworkers	Digital competence means daring and challenging oneself as a nurse	New competence fostered the courage to challenge oneself	Feeling strengthened by new knowledge	Being open to change and new ways of working
But [there could be] something as small as not being able to log into the computer. If something goes wrong and there’s a hold-up, then you’re stranded	Nurses struggled with computers and lacked support	Frustration and need for support and equipment	Struggling to manage the technology	Struggling to handle requirements
Absolutely, [the pandemic] has been the engine to get [digitalisation] started. Before, we had just talked about it, but then everything happened so quickly. … It was a very sudden change that needed to be made to maintain care and contact. As a result, we increased our flows during the pandemic and even had shorter wait times	Need for quick adjustment to maintain good care contact	External circumstances a driving force for developing changes in work	The pandemic as a driving force for change	Developing new ways of working
Last week, I had a patient, and she was interested in digital meetings and said she would prefer to sit at home	Patient interested in digital meetings and wanted to stay at home	Enabling patients to make their own choices	Initiating individually tailored meetings	Focusing on patients despite the distance

### Ethical considerations

The study was conducted following the Declaration of Helsinki [24]. Before the interviews commenced, the participants received oral and written information outlining the study’s purpose and were guaranteed confidentiality in the presentation of the findings. They were also informed that their participation was voluntary and that they could withdraw from the study at any time without needing to provide an explanation. All participants gave their written informed consent to participate in the study before the data collection. The study was assessed by the Swedish Ethical Review Agency in Umeå, Sweden (Dnr: 2019–03,353) and conducted according to the ethical principles recommended by the Research Council.

## Results

**Fig. 1:**
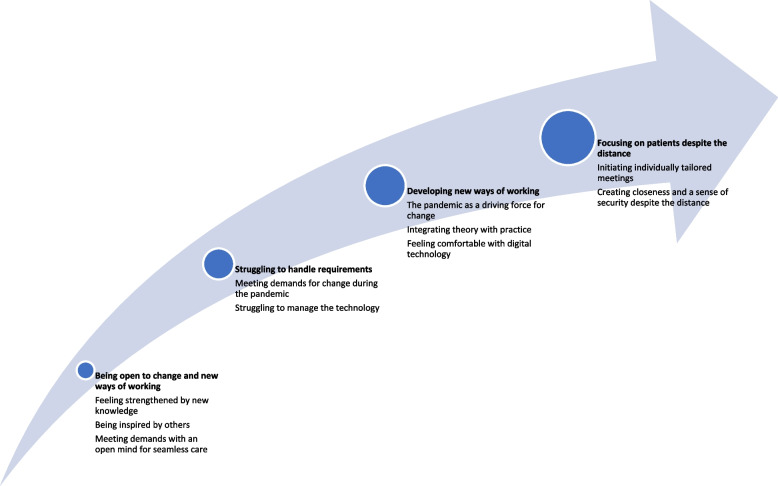
Illustrating the result as a process of development based on RNs experiences with PCC and competence after participating in a course in DCC

The comprehensive understanding formed from analysis afforded a compound picture of the RNs’ experiences with PCC and competence after participating in a course in DCC revealed categories with subcategories that was woven together and interpreted in light of relevant theory and literature. The four categories: being open to change and new ways of working; struggling to handle requirements; developing new ways of working; and focusing on patients despite the distance was described as a process of development for the RNs and is shown in in Fig. 1. The results are presented below with quotations from the nurses interviewed (RN 1–8 and Interview, I, 1 or 2).

### Being open to change and new ways of working

#### Feeling strengthened by new knowledge

Increased knowledge from the DCC course empowered RNs, informed their arguments for promoting digital care and broadened both their perspective on and their understanding of the complexity of their profession. Gaining knowledge helped them articulate the practical work that they regularly perform, which strengthened their professional role. The RNs described how their new knowledge sparked curiosity and emboldened them to find new ways of using digital care. Their participation in the DCC course with practical training contributed to their positive attitude, while their increased digital competence generated opportunities for better resource utilisation and, by extension, benefits for the patients. The new-found knowledge prepared the RNs to promote technical development based on patients and their care needs of care and patients instead of becoming passive recipients of technology.

Digital competence involves daring to deal with new digital systems and to challenge myself and [my] coworkers. (RN 2, I 1)

#### Being inspired by others

The RNs reported feeling inspired by the other RNs’ experiences with digital care meetings and had created professional networks where they honed their understanding that implementing digital care meetings relates to competence and self-image and the need for social interaction. The RNs felt encouraged by praise from managers and by the resources made available to enable them to revise their nursing practice, which inspired them to further develop digital care meetings. Via the managers’ network in development and management, the RNs dared to integrate digital care into their clinical activities, which drove the work forward.

We’re on a digital train moving forward fast, and we’ve only just started. (RN 7, I 1)

#### Meeting demands with an open mind for seamless care

Participants described how the demand for digital care had gradually increased among patients and various health care professionals alike. Patients wanted to send digital images instead of engaging in time-consuming physical visits. Collaborations had developed in which patients could remotely contact treatment units in specialised health care via equipment set up at health centres, which had been especially valuable for patients in sparsely populated areas. The lack of computer systems and routines for handling the patients’ wishes, however, was challenging for the RNs. The demand for joint staff training between regionally and municipally employed RNs increased, and such training soon commenced digitally. As a result, the RNs experienced increased medical quality such that patients could receive seamless care at any hour of the day.

It’s not just the care that should benefit; the patient should always benefit, too. We should not rationalise everything, but we must offer opportunities to the patient. (RN 5, I 1)

## Struggling to handle requirements

### Meeting demands for change during the pandemic

When the pandemic suddenly increased the demand for a rapid digital transformation, neither RNs nor health care systems were prepared for such an abrupt change. Having only limited experience with digital care meetings, the RNs had to rapidly adapt to the situation. They had lacked guidelines, which led alternative solutions to emerge, and expressed relief that the pandemic had contributed to relatively straightforward decision-making processes focused on implementing digital care meetings. Whereas the RNs had previously only discussed digital care, their concrete digital work had been accelerated by the pandemic and thus turned into a reality. In parallel, their health care organisation had become solution-focused, which had made it easier for them to individualise their work with patients.

We found new ways [of working], and we’ll never return to the old ways. (RN 1; I1)

### Struggling to manage the technology

RNs described struggling with digital technology and needing various amounts of technical and practical support before feeling comfortable with digital care meetings. Technical systems had been perceived as being sluggish, time-consuming, and irritating, and the RNs described how they had previously been denied access to various digital systems when logging in, which rendered digital care meetings impossible. They described feeling insecure and inadequate when patients experienced technical difficulties and expected practical help from them during digital care meetings. RNs thus needed to increase their technical skills and described seeking constructive solutions and rehearsing the use of digital technology to gain mastery. However, they also described feeling more creative when working digitally, and their positive attitude motivated them to continue working that way. They estimated spending between 25 and 100% of their working hours using different digital health care systems. Their narratives about digital care meetings focused on technical barriers and the lack of equipment, including headphones, which had resulted in frustration when they had to start their workdays by searching for equipment.

But [there could be] something as small as not being able to log in to the computer. If something goes wrong and there’s a hold-up, then you’re stranded. (RN 4; I1)

## Developing new ways of working

### The pandemic as a driving force for change

The pandemic was described as driving the implementation of digital care meetings, which became a prerequisite for maintaining contact with patients. Over time, the pandemic has continued to alter the RNs’ working methods, including starting to receive patients digitally, which lessened wait times. The RNs described this as a positive experience but also as creating stress, for it led to clear shortcomings in health care.

Absolutely, [the pandemic] has been the engine to get [digitalisation] started. Before, we had just talked about it, but then everything happened so quickly. … It was a very sudden change that needed to be made to maintain care and contact. As a result, we increased our flows during the pandemic and even had shorter wait times. (RN 8, I 2)

### Integrating theory with practice

More theoretical knowledge from the DCC course fuelled the RNs’ interest in digital care meetings, which contributed to new knowledge, inspiration, and development in their clinical practice. In turn, the practical use of digital care meetings increasingly developed once the DCC course had assured the RNs of their role in developing digital care. As a result, the RNs reported now being invited to contribute to decisions regarding the development of digital care. The RNs also described how they now understood digital care meetings more profoundly and felt more comfortable creating new models to test out. Specifically, the RNs gained an increased professional understanding of abstract theory by connecting it to practical applications, which generated working methods with a scientific basis.

I think that when you read or take courses, the combination of theory and discussions enriches your learning a lot, and then you try to apply [the new knowledge] in real life to see whether it works. That’s inspiring. (RN 5, I 2)

### Feeling comfortable with digital technology

The RNs described needing to feel comfortable with the technical equipment to focus on the patients, not the technology. They described accepting that it would take time to learn to use the new technology and how mental barriers and technical difficulties had been remedied with increased experience and technical competence. To increase their familiarity with the patient and mitigate stress, the time required for a digital care meeting needed to be equal to the time required for a physical meeting. The RNs described feeling assured when digital workstations functioned as intended and when they were offered an individual workroom with the opportunity to work undisturbed. They reported that their technical interest and courage in using digital care solutions increased their know-how and supported their drive to find user-friendly technical systems in which dual monitors facilitated work in parallel computer systems.

We need to have education and to be good at educating ourselves so that we feel familiar with technology. (RN 6, I 2)

## Focusing on patients despite the distance

### Initiating individually tailored meetings

By being sensitive to patients’ needs, the RNs adapted care on a patient-by-patient basis and, in turn, increased their ability to assess which patients digital care meetings were suitable for. In that way, patients could influence their care and be supported through digital care meetings. RNs described how they had previously directed the care, but patients were now being increasingly included in decision-making related to their care. RNs described adapting digital care meetings by being creative and solution-oriented based on the patients’ resources. Moreover, by lending new technical equipment to patients with outdated or no equipment, the nurses reduced the risk of excluding patients.

Last week I met a patient in palliative care, and she was interested in digital meetings and said she would prefer to sit at home with her family. So, I arranged a digital care meeting for her. You need to take time to think: for whom is this an option? And when does it fit? [No solution] can be a generalisation. (RN1, I 2)

### Creating closeness and a sense of security despite the distance

The RNs reported how they worked with closeness in digital meetings and this increased patients’ sense of security in digital meetings. Capturing the patients’ gaze in the camera especially increased the feeling of being present. The RNs described how moving images made it easier to create relationships than it is via the telephone because patients could see a face, which also contributed to the RNs’ perception of the nuances in the conversation and body language despite the distance. They also described how they reflected on how patients perceived them, which heightened their understanding of digital care meetings and facilitated the creation of security. For the RNs, the experience of having contact, even if remotely, was able to bridge the distance between provider and patient. For example, during their conversations, some RNs and patients simultaneously placed their palms on their respective screens. The sense of proximity and openness increased if the RNs had already met the patients during physical visits.

The digital care meeting is different and perhaps more impersonal in some way [than physical meetings], and so it’s important to try and make it as personal as possible …one way is to reach out and place a hand on the screen. It’s physical contact but still not. (RN 1, I 2)

### Cooperating for safety in health care

Despite the geographical difference, RNs reported that digital care meetings were found to have fostered cooperation among professionals and increased proximity in caring for patients. The RNs reported the availability and flexibility of care had increased so that patients could receive care despite the pandemic. Digital cooperation was found to have increased the availability of care for residents in sparsely populated areas and to have reduced home care services and ambulance trips. Collaboration thus meant that patients could continue to be cared for at home and that long-distance ambulance rides could be avoided. The RNs also found that digital care meetings were suitable for difficult conversations about palliative care and invited relatives to support patients, just as they did during physical visits. The digital solution thus reduced necessary travel and enabled patients to continue to be cared for at home despite facing advanced illness.

You sometimes must be a magician and an inventor when you work in sparsely populated areas. You want to avoid transporting an old sick person 180 km to the hospital if it’s not necessary. (RN 7, I 2)

## Discussion

The understanding and knowledge gained from the analysis show how RNs’ experienced PCC and how their competence in digital care expanded as a process of development, following the DCC course. Authentic training during the DCC course created opportunities for the RNs to develop new ways of working and contributed to their know-how about clinical activities and, in turn, their confidence and openness. The transition from physical to more digital care meetings, accelerated by the COVID-19 pandemic as a means of maintaining patient contact, altered their work situations and contributed to common care benefits. The rapid introduction also contributed to the RNs’ uncertainty and highlighted their technical inability to support patients at digital care meetings. Increased digital competence, however, created a sense of security that translated into clinical know-how, which encouraged the RNs to increasingly invite more patients to attend digital care meetings and allow a wider range of reasons eligible for such visits. By extension, the RNs’ increased knowledge and sense of security sharpened their skills, especially with support from managers.

In light of the aim to describe RNs experiences with PCC and competence after participating in the DCC course, the results revealed that RNs’ theoretical knowledge and digital competence via reflections with others advanced from novice to expert [25] and the RNs changed from being technically inept to being able to concretely help colleagues and patients with digital care meetings. The RNs described that the ongoing pandemic had contributed to that development and accelerated the introduction of digital care meetings into their work, and the results indicate how inexperience with digital care meetings in combination with rapid change had left the RNs unprepared for their new professional situation. This situation was characterised by fears and anxieties that initially focused on technical obstacles. Studies have indicated that digital technology in care changes the role of RNs, which can both reduce and create stress for RNs [26, 27]. At the same time, RNs play a significant role in supporting patients’ use of digital technology, an activity in which a lack of support pushes patients away [28–30]. Taken together, the evidence suggests that RNs are increasingly embracing a professional role that involves using digital technology and necessitates conditions that broaden their area of knowledge. According to Shepard-Law et al. [31], knowledge develops from theory via reflection about one’s and others’ experiences. In parallel, the opportunity to identify oneself as a novice in a new work situation despite having another level of knowledge in a different domain increases one’s sense of security [25, 32, 33]. Benner [25, 33] described how having experience and expertise in certain areas makes it easier to be a novice in new tasks where previous experience is lacking. However, according to Friesen-Storms et al. [34], ignorance and negative attitudes are barriers to integrating knowledge into clinical practice. Accordingly, education across one’s professional life is crucial to developing competence via a reflexive approach, which requires understanding one’s opportunities for development and being open to new ways of working. However, new ways of working require new skills such as listening and developing a “clinical hearing” [35]. Boström et al. [36] shows RNs communication with patients when practicing PCC at distance over the phone, and how RNs through “careful listening” created space for the individual patients to express their thoughts and feelings and emphasized each patient’s capabilities and resources. The RNs also gained an understanding of PCC and what it means to patients and to themselves as practitioners. This is also in line with our results that show that interactions in care meetings shifted from depending on physical proximity to building proximity despite the distance. Although they had limited experience with digital care meetings, the RNs had extensive experience with physical care visits, which helped them develop the ability to remotely ensure a feeling of closeness in the care relationship with patients. According to Ekman et al. [2], PCC based on joint reconciliation requires a documented care plan, involves the patient, and increases the patient’s participation. In contrast, patients who are not treated based on their own perceptions often continue to seek care [37]. These findings are congruent with Epstein and Street [38], namely, that PCC meetings require knowledge, the ability to focus on the patient, and an understanding of the patient’s experiences and needs. Such a conscious presence is created via reciprocity, communication, and interaction [39, 40]. Benner [41] added that RNs’ perceptual ability and judgement based on each step in developing competencies result from experiential learning acquired from meetings with patients. This dynamic suggests that developing experience, compassion, and support enables good nursing and PCC regardless of whether care meetings occur in person or digitally.

In our study, the digital care meetings allowed the RNs to reduce their travel time and home visits, which increased the number of digital patients visits they could make and provided a more flexible workday. Additionally, the patients’ reduced travel time to care facilities also increased their independence and access to care. Benner [33] observed that when RNs collaborate and integrate their work teams into their strategies for efficiency, patients’ treatment benefits. In another work, Morley and Cashell [42] found that collaboration increases the efficiency and the quality of care, while Wilkes et al. [43] highlighted that digital cooperation benefits from a clear division of roles and in turn creates socioeconomic benefits. Such observations suggest that digital care meetings, which presuppose cooperation and organisational adaptation, provide better resource utilisation in the form of increased care contact. As a result, the quality of care improves, and sustainable socioeconomic benefits with the distribution of human and monetary resources become possible.

Our results also showcase that the RNs were accustomed to centring care around their patients. Thus, that trend may indicate that care meetings risk becoming imbalanced to the point where patients feel dependent upon the provider. Research has shown that increased accessibility to care contributes to equal care and benefits from RNs’ ability to integrate technology and refine their working methods [44–46]. These results suggest that a patient’s dependency decreases with transparency but increases with digital access to care.

Last, our results highlight the importance of managers in developing new working methods by providing the necessary financial resources and inspiring professional development. According to Benner [25, 33], a lack of recognition from managers can lead to difficulties for RNs in understanding their professional role and professional performance. This dynamic aligns with the conclusions of Huy [47] that managers are members of organisations capable of relieving the anxiety that staff may feel during processes of change. At the same time, Carlström [48] described how managers in health care often have experience as health care providers themselves but lack managerial training and thus identify with staff and patients, which risks a conflict of loyalty when they face bureaucracy and questions about financial resources. Other research has shown that an organisation’s adaptation to change depends on managers’ ability to foster participation and a supportive culture [49, 50].

### Methodological limitations

To achieve trustworthiness in research, the authors have strived to provide sufficient information on the research process. Direct quotations from the interviews increase readers’ ability to judge the credibility of the study. Confirmability can be established by returning to the interview texts throughout the research process as described. The authors had extensive experience as RNs themselves and continuously discussed the analytical process to strengthen the credibility and trustworthiness of the research [17, 23], they also had previous experience with conducting qualitative research. Furthermore, there were no prior relationship to the participants. The eight participants were interviewed twice, and the relatively small sample was compensated by rich, nuanced interviews. The sample size does not necessarily matter when a study’s results are the sum of the richness of the collected material, which can be increased by conducting longer, more in-depth interviews [17]. The trustworthiness of studies increases with a sample showing variation in age, gender, context, and experience [17, 23, 51]. The sample consisted only of women from primary healthcare, and even if such homogeneity reflects the gender distribution of RNs in Sweden, it could nevertheless be a limitation. As with most qualitative research, the results should be considered in the contexts where they emerged and cannot necessarily be generalised to other settings or screenings. Some researchers criticise the inductive and descriptive approaches for summarising empirical data without providing new insights. However, the results contribute to a new understanding and knowledge of how RNs experience PCC in digital care meetings and how RNs competence expanded as a process of development following education in digital competence [17, 23, 52, 53].

## Conclusions

Our study has described how RNs’ experience and competence in digital care meetings changed and developed after participating in the DCC course. Training in both practice and theory increased RNs’ competence and facilitated the creation of new knowledge. The RNs’ professional role was strengthened by taking part of changing of work routines. Digital care meetings were shown to be distance bridging and complementary to physical care meetings and to contribute to PCC by making health care more accessible and promoting patients’ participation in their care.

Because the increased availability of health care via digital means has affected the consumption of care, RNs need of lifelong tailored education must be met by nursing education programs. Although digital care is accessible, efficient and enables care regardless of geographical conditions, its innovative development needs to be based on science and experience and RNs are key personnel in this process.

## Supplementary Information

**Additional file 1. **A checklist of items that should be included in reports of qualitative research. You must report the page number in your manuscript where you consider each of the items listed in the checklist. If you have not included this information, either revise your manuscript accordingly before submitting or note N/A.

## Data Availability

The datasets generated and analysed (i.e., transcribed interviews) during the current study are not publicly available due to the raw data containing information that could compromise the privacy of the research participants and due to the fact that participants of this study did not agree for their data to be shared publicly, but are available from the corresponding author. upon reasonable request.

## Abbreviations

PCC: Person-centred care
DCC course: Digital Competence in Care course
BYOD1: Bring-Your-Own-Data
BYOD2: Bring-Your-Own-Device
COREQ: The COnsolidated criteria for REporting Qualitative research checklist
